# Exploring the Use of Mobile Health to Improve Community-Based Health and Nutrition Service Utilization in the Hills of Nepal: Qualitative Study

**DOI:** 10.2196/17659

**Published:** 2020-09-11

**Authors:** Ajay Acharya, Kenda Cunningham, Shraddha Manandhar, Niva Shrestha, Mario Chen, Amy Weissman

**Affiliations:** 1 Family Health International 360 Kathmandu Nepal; 2 Helen Keller International Kathmandu Nepal; 3 Global Health, Population, and Nutrition Family Health International 360 Durham, NC United States; 4 Asia Pacific Regional Office Family Health International 360 Bangkok Thailand

**Keywords:** mobile health, text messages, SMS text message, qualitative study, Nepal, health and nutrition services, health mothers’ group, female community health volunteers, mobile phone

## Abstract

**Background:**

With mobile phone coverage and ownership expanding globally, mobile health (mHealth) interventions are increasingly being used to improve coverage and quality of health and nutrition services. However, evidence on mHealth intervention feasibility and factors to consider during program design is limited in low- and middle-income countries like Nepal.

**Objective:**

This study aimed to examine the potential of using text messages to improve health and nutrition services by exploring mobile phone ownership and sharing; mobile phone use and skills; and interest, preferences, and limitations regarding mHealth interventions.

**Methods:**

We conducted 35 in-depth interviews with 1000-day women (the period from conception to a child’s second birthday), health facility staff, and female community health volunteers (FCHVs), as well as eight focus group discussions with health facility staff, FCHVs, and 1000-day household decision-makers (ie, husbands, mothers-in-law, and fathers-in-law). We also conducted a mobile phone skills test. We employed thematic analysis using framework matrices and analytical memos.

**Results:**

The study included 70 study participants, of whom 68 (97%) had a mobile phone, and phone sharing was uncommon. Use of text messages was most commonly reported by 1000-day women and health facility staff than household decision-makers and FCHVs. More than 8 in 10 participants (54/64, 84%) could dial numbers, and the majority (28/34, 82%) of 1000-day women, health facility staff, and male decision-makers could also read and write text messages. We found that 1000-day women preferred educational and reminder messages, whereas health facility staff and FCHVs desired educational and motivational messages. Participants suggested different types of texts for 1000-day women, families, FCHVs, and health facility staff, and reported less value for texts received from unknown phone numbers.

**Conclusions:**

A text message–based mHealth intervention is acceptable in the hills of Nepal and has the potential to improve community health and nutrition service utilization, particularly by sending meeting reminders and by providing information. Our findings contribute to text message–based mHealth intervention design in under-resourced settings.

## Introduction

Mobile health (mHealth) involving the use of mobile phone technologies, such as text messaging, voice services, global navigation satellite systems, and mobile apps, is increasingly being adopted as a means of delivering public health interventions [1]. With expanding mobile phone ownership and accessibility in low- and middle-income countries (LMICs), interest in using mHealth interventions to overcome some of the health service delivery barriers has grown in recent years [1,2].

Text messages delivering reminders and other information can encourage recipients to adopt or maintain healthy behaviors. A previous meta-analysis showed an increase in antenatal care (ANC) visits and delivery by skilled birth attendants among women who received text message reminders about ANC appointments and health information during their pregnancy [3]. Despite the increased use of mHealth to improve health service uptake in LMICs [4], little is known about its feasibility and effectiveness. Although systematic reviews of mHealth in LMICs showed positive effects on maternal and child health service utilization [5-8], the reviewed studies mostly employed text messaging and varied widely in intervention and implementation details, study design, and context. Further, a systematic review on the use of mHealth for behavior change communication (BCC) interventions showed no promising results regarding effectiveness [9]. All of these systematic reviews identified the need to continue building the mHealth evidence base and highlighted the specific need for high-quality implementation research [5-8].

In LMICs like Nepal, where there is nearly universal mobile phone ownership and coverage of mobile phone service, the potential for mHealth interventions to extend the reach and quality of health services is encouraging. As of 2016, nearly all Nepali households (93%) owned at least one mobile phone (94% in urban areas, 91% in rural areas). Among 20 to 24-year-old individuals (the age group most likely to possess a mobile phone), 85% of women and 96% of men owned a mobile phone [10]. In Nepal, prepaid service, where customers pay before accessing the service offered by two leading mobile network service providers (Nepal Telecom and Ncell), is common.

Despite measurable progress in health and nutrition outcomes in the last few decades, Nepal retains the highest rates of maternal mortality in South-East Asia [11], and 36% of children under 5 years are chronically malnourished (stunted) [10]. The reasons for this include poor health service delivery, with financial constraints, unequal distribution of the health workforce, challenges in service extension and integration, and poor access to and inconsistent quality of health services [12,13].

Many development partners and the government of Nepal are committed to improving the availability and uptake of quality health services. For instance, Helen Keller International and Family Health International (FHI) 360 (authors’ organizations) and five other partners are implementing *Suaahara II* (2016-2021), a United States Agency for International Development–funded multisector nutrition program, which is now operational in 42 of Nepal’s 77 districts. *Suaahara II* involves both household and community-level interventions to improve health and nutrition-related behaviors and increase demand for quality health services, as well as system-level interventions to improve the quality of health services both at facilities and in communities [14].

Believing that mHealth interventions may help address some of the most challenging structural barriers to accessing community health and nutrition services [15], development partners and the government are beginning to implement mHealth activities in Nepal. For example, in January 2018, *Suaahara II* started an age- and stage-specific text message campaign of reminders and motivational messages targeting 1000-day households (the period from conception to a child’s second birthday), which involves messages to improve health and nutrition practices, as a new social and behavior change intervention to complement its ongoing home visits, group meetings, food demonstrations, and mass media radio programs. This campaign is based on monitoring data pointing to the high prevalence of mobile phone ownership and use [16] and two rounds of qualitative formative research exploring the potential for sending text messages to provide information on health and nutrition. *Suaahara II* has also been interested in designing a similar text message intervention for service providers to help address supply-side health service constraints.

Evidence on the feasibility of using text messages to improve uptake of government-led health and nutrition services in LMICs, however, is scant. Given these gaps, we conducted a qualitative study with both 1000-day households and service providers to determine the feasibility of using mHealth within the context of *Suaahara II* to improve the reach and quality of health and nutrition services in Nepal. Specifically, this study aimed to understand the potential of using text messages with 1000-day households and/or service providers to improve health and nutrition services by exploring mobile phone ownership and sharing; mobile phone use and skills; and interest, preferences, and limitations regarding mHealth interventions.

## Methods

We conducted a qualitative study employing in-depth interviews (IDIs) and focus group discussions (FGDs) with health facility staff, female community health volunteers (FCHVs), 1000-day women, and other household decision-makers (ie, husbands, mothers-in-law, and fathers-in-law of the 1000-day women). The study was conducted in December 2018 in Syangja, a hilly district of Nepal, comprising 11 urban and rural municipalities made up of 97 wards with a population of about 290,000. The district has 97 public health facilities and 612 FCHVs [17]. We purposefully selected Syangja because it is a *Suaahara II* district, is geographically similar to other hilly districts, and has good mobile network coverage, a prerequisite for a text message intervention.

Within Syangja, the research team purposively selected one rural (Kaligandaki) and one urban (Chapakot) municipality. In consultation with *Suaahara II* staff and based on the implementation status of service-related interventions, the team selected one ward in each municipality. Kaligandaki’s sampled ward had implemented the Self Applied Technique for Quality Health (SATH) approach, a *Suaahara II* participatory approach that supports health mothers’ groups (HMGs) to conduct live social mapping with community members, encourages mothers to seek health services, and tracks 1000-day women’s health service utilization, whereas Chapakot’s sampled ward had not received this intervention.

All health facility staff and FCHVs of the selected wards were included in the study. Families of women in the 1000-day period were purposively selected. To identify 1000-day women, each health facility in charge selected one more and one less effective FCHV (performance assessed via monthly reports and evaluations) who in turn provided the names of five 1000-day women from their FCHV-led monthly HMG meeting registry. We asked FCHVs to ensure selection of mothers with diverse backgrounds, including different caste/ethnicity groups, age, level of education, and mobile phone access. Five additional 1000-day women per municipality were recruited by asking the five selected 1000-day women to each refer one additional mother from the community. Researchers also invited other 1000-day family members to participate in the study by asking each 1000-day woman who completed the interview to identify all adult decision-makers (husband, mother-in-law, or father-in-law) currently living in the same household and available to participate in the study.

We conducted 9-day training for 10 data collectors and pretested all data collection tools. The trained data collectors conducted IDIs and FGDs. IDIs were conducted with 10 FCHVs (five per ward) and 20 1000-day women (10 per ward). Eight FGDs were conducted as follows: four with male and female household decision-makers separately (two from each ward), two with FCHVs (one from each ward), and two with health facility staff (one from each ward). Researchers perceived this sample size to be adequate to reach saturation [18] but agreed to continue data collection until saturation was achieved. No new themes emerged in the final IDIs or FGDs.

Topics explored during IDIs included access to a mobile phone; phone and network coverage quality; and phone and text messaging use and preferences such as timing, type, frequency, recipient, and intention to reply to or share the text messages received. Similarly, wider community perception on mobile phone use/SIM card change, network access, phone sharing, text message information sharing with others, text message interest and preferences, and thoughts on using text messages as a BCC intervention were explored during FGDs.

Researchers also assessed the ability of all 1000-day women, FCHVs, and health facility staff to use a mobile phone. First, researchers asked the participants if they could dial on the phone and read and/or write a text message. Thereafter, researchers asked the participants to dial a phone number (usually that of the researcher) and then to read any existing text message on their mobile phone. If there were no text messages on the phone, the researcher sent a sample message in Nepali and asked the participant to open and read it. The researcher also asked each participant to write and send a text message from their phone to the study moderator’s phone number.

IDIs and FGDs were audio-recorded and then transcribed into Nepali by trained data collectors. Two translators were hired to translate the transcripts into English. Two researchers selected and independently open coded the same eight transcripts using NVivo 12 (QSR International). These were then used to develop the initial themes and codebook, which were used to code the remaining transcripts. On occasion, researchers added new themes and codes generated during coding. The two researchers developed a framework matrix using NVivo and independently analyzed the data producing analytical memos and then triangulated the results. Themes were identified and analyzed, and any similarities/differences among data collection sites and as per the categories of participants were noted. Following this round of analysis, in July 2019, researchers visited the study sites and gathered all of the health facility staff and FCHVs who had been study participants to share and validate the emerging findings and explore their perceptions and agreement/disagreement vis-a-vis the findings related to 1000-day families.

The study was approved by the Nepal Health Research Council on July 2, 2018, and FHI 360’s Protection of Human Subject Committee on November 21, 2018. All study participants provided written informed consent for both the interview/discussion and recording.

## Results

### Demographics

We conducted 35 IDIs and 8 FGDs, with a total of 70 participants (Table 1). The majority of health facility staff were young (mean age 28 years), and 5 out of 8 (63%) were from the Janajati socially disadvantaged caste in Nepal, with some Brahmins (socially advantaged caste). All the health workers possessed the qualifications required for their positions. Their service duration ranged from just 4 days to more than 15 years. The majority of FCHVs were in their early fifties (mean age 51 years), and 11 out of 17 (65%) were Brahmins, with some Janajatis. While slightly more than one-third (6/17, 35%) of FCHVs had completed 10 years of schooling, 53% (9/17) had not completed secondary school (below 8 years of schooling), and 2 out of 17 (12%) had no formal education. Nearly all had been FCHVs for more than 16 years, but two FCHVs were appointed only 4 months before data collection.

**Table 1:** Distribution of study participants.

Approach		Value, n		Description	
**In-depth interviews**		35			
	Health facility staff		6		Three per site
	FCHVs^a^		9		Four in Kaligandaki and five in Chapakot
	1000-day women^b^		20		10 per site
**Focus group discussions**		8			
	Health facility staff		2		One per site with four participants per FGD^c^
	FCHVs		2		One per site with four participants per FGD
	Male decision-makers		2		One per site with four to five participants per FGD
	Female decision-makers		2		One per site with five participants per FGD

^a^FCHVs: female community health volunteers.

^b^The period from conception to a child’s second birthday.

^c^FGD: focus group discussion.

Most 1000-day women were in their mid-twenties. Of the 20 1000-day women, 35% (7/20) had completed at least 10 years of schooling, and 4 out of 20 (20%) had not completed primary school. The majority (14/20, 70%) were Brahmins, with some Janajatis and Dalits. Most women were living in a joint family with their in-laws. All female decision-makers who participated in the study were mothers-in-law (n=10), with a mean age of 50 years. The majority of male decision-makers in the study were husbands (n=6), with a mean age of 29 years. A few decision-makers were fathers-in-law (n=3), with a mean age of 55 years.

### Mobile Phone Ownership and Sharing

All study participants, except two of the 10 mothers-in-law, had a mobile phone and reported high mobile phone ownership among adult family members. The majority (48/64, 75%) of participants had a smartphone. We found no differences in phone ownership by age, sex, or caste among the majority of study participants. A few participants mentioned that ownership among women has increased to enable them to contact male relatives, many of whom emigrate for work.

The majority of the 1000-day women reported that they are allowed to access and read text messages, and most decision-makers confirmed this lack of restriction. Phone sharing was uncommon at the study sites, with a few exceptions. The majority (15/19, 79%) of 1000-day women mentioned accessing another phone, mainly that of their husbands, to make urgent calls when their phones had insufficient credit, when their phones stopped working, or when wanting to watch videos or check Facebook and this was not possible on their own phones. Some husbands also mentioned using the same phone as their wives (1000-day women). Some mothers-in-law expressed hesitation to ask their daughters-in-law to use their phones but felt comfortable sharing their phones with their daughters-in-law.

Being out of network coverage was uncommon at the study sites. The majority of study participants reported never having changed the SIM card. In the FGD with Kaligandaki health facility staff, age differences in SIM card behaviors were reported as follows:

The aged people use the old number; we find the newer generation changing the SIM frequently.

Health facility staff, Kaligandaki, FGD

### Mobile Phone Use and Skills

Participants reported using mobile phones for various purposes, but primarily to receive calls. Text messaging was more frequently reported by 1000-day women and health facility staff compared with other male and female decision-makers and FCHVs. The majority (14/20, 70%) of 1000-day women reported using their phones to access the internet, particularly Facebook, Imo (video call and chat app), and YouTube, to make video calls, chat via text/messenger, and play games. Nearly all health facility staff, but only few FCHVs, reported accessing the internet on their phones. Some participants also reported differences in phone use among their household members, with fathers-in-law and mothers-in-law using the phone primarily to make calls and younger family members (eg, mothers and fathers of young children) using the phone to access the internet and for messaging.

More than 8 in 10 study participants (54/64, 84%) could dial phone numbers, but only about 72% (46/64) were able to read and 56% (36/64) were able to write text messages. The majority of those who could read and write text messages were 1000-day women, their husbands, and health facility staff (Table 2). Five of the 10 FCHVs reported a preference for voice compared to text messages, stating that they prefer to listen than read. This was supported by the mobile skills test, which found that three of the 10 were illiterate. Some of the FCHVs and health facility staff reported a preference for receiving important messages via both voice and text. In a health facility staff FGD, participants reported that text messages would be fine for health workers because of their capacity to read; however, a combined form would be more effective because reading would be bothersome especially when text messages are long. One health facility staff member made the following statement:

Person may feel bored in the middle of messages and feel why should I read this. So, it would be effective to listen, as well. The things that someone listened to, that person can present it in another place.

Health facility staff, Kaligandaki, FGD

**Table 2:** Mobile phone use skills.

Category	Phone dial^a^	SMS text message writing and reading^a^
1000-day women^b^ (N=20)	20 (100%) could dial the phone number	16 (80%) could read and 14 (70%) could write text messages in Nepali
FCHVs^c^ (N=17)	15 (88%) could dial the phone number	14 (82%) could read and 8 (47%) could write text messages in Nepali
Health facility staff (N=8)	8 (100%) could dial the phone number	8 (100%) could read and write text messages in both Nepali and English
Male decision-makers (husbands) (N=6)	6 (100%) could dial the phone number	6 (100%) could read and write text messages in Nepali
Male decision-makers (fathers-in-law)^d^ (N=3)	3 (100%) could dial the phone number	1 (33%) replied that they could read text messages in Nepali
Female decision-makers (mothers-in-law)^d^ (N=10)	2 (20%) could dial the phone number	1 (10%) replied that they could read text messages in Nepali

^a^Data are provided as n (%).

^b^The period from conception to a child’s second birthday.

^c^FCHVs: female community health volunteers.

^d^Assessment based on response.

### Interest and Preferences for Text Message Interventions

Nearly all participants expressed interest in receiving text messages with health and nutrition information and about community-provided services, such as HMG meetings. Some topics of interest mentioned were nutrition, timing of ANC visits, postnatal care checkups, child immunization, growth monitoring and promotion, family planning, deworming, iron and calcium consumption, HMG meeting times and discussion topics, drinking water purification, and personal, family, and environmental sanitation.

Many 1000-day women also mentioned a desire for messages on the topics discussed in the HMG meeting to help participants retain information. The 1000-day women also expressed an interest in receiving text messages because the content would give them something to discuss when they are with their peers. Women made the following statements:

Some things could be confused or forgotten or due to noise could not be understood properly in the meetings. We can get information about that thing by reading [a] message.

1000-day woman, Chapakot, IDI

SMS is good in that we can read the message, we talk about the message we read, and we can talk to each other when we go to cut grass and leaves.

1000-day woman, Kaligandaki, IDI

Health facility staff and FCHVs were also interested in health and nutrition-related text messages and perceived them as a type of job aid. They explained that the new information would update and/or refresh their knowledge, and they would be able to store and access the information repeatedly on their phones. They also mentioned a desire for motivational messages that would inspire them to improve their work, as described by one IDI participant as follows:

If messages are sent regarding how a health worker should work, we will have to think about those things. We will realize if we are out of track, the things we need to improve. I will get a chance to improve those things.

Health facility staff, Kaligandaki, IDI

Health facility staff and FCHVs also highlighted that text messages to families may encourage greater HMG participation (for example, by reminding mothers of the meeting time and communicating with mothers who they are unable to reach because of their heavy workloads). Health facility staff made the following statements:

If people get [a] reminder message today about tomorrow’s meeting, then they can show that message to the household head and tell [them] that they have to go tomorrow.

Health facility staff, Chapakot, IDI

If the message goes to the family, there can be family discussions about the meeting, which might help create a more convenient environment within the family to allow women to attend meetings.

Health facility staff, Chapakot, FGD

Health facility staff also noted that such messages would provide information that would tempt women to join HMGs to learn more and improve women’s perception of the importance of HMG meetings, thus encouraging more active participation. One health facility staff member made the following statement:

If topics of discussion are sent through SMS, they will realize how appropriate is it for them to know about it. There might be some things they might have known and some that they might not have known. They will develop a feeling that they should go.

Health facility staff, Kaligandaki, IDI

Health facility staff from both sites suggested that text messages should also be sent to FCHVs because they are the main organizers of the HMG meetings and the text messages can serve as a reminder and a guide in meeting preparation. FCHVs in Chapakot identified health facility staff as the priority text message recipients so health facility staff can teach the FCHVs new information learned from the messages. FCHVs in Kaligandaki prioritized health facility staff and FCHVs themselves but recommended different content for each group, since health facility staff are perceived to have more knowledge than FCHVs. Health facility staff in Chapakot noted that 1000-day mothers are the most crucial text message target.

Most FCHVs and household decision-makers from both study sites prioritized 1000-day women as primary text message recipients. This preference was because the women in the 1000-day period were perceived to be more interested in these matters, more educated, and more able to use the information as they were considered less likely to migrate away for work.

Some participants in both IDIs and FGDs expressed preferences regarding the frequency and timing of text messages. For example, in both settings, the majority (18/20, 90%) suggested that it would be most appropriate to receive a reminder text message 1 to 2 days before the HMG meeting. Furthermore, the majority (17/20, 85%) of 1000-day women preferred receiving text messages in the evening, but before 8 PM. Some women, however, reported being comfortable receiving text messages any time during the day. The majority (19/20, 95%) suggested that an SMS campaign send text messages once a month. One woman made the following statement:

Instead of making people feel bored by sending many messages, it would be good to send once or twice a month. Many messages should not be sent.

1000-day woman, Chapakot, IDI

When asked about their response to receiving text messages, the majority (17/24, 71%) reported that they would read a message from an unknown phone number out of curiosity. Yet, some participants, particularly FCHVs and 1000-day mothers, mentioned that they would not read it, delete it, or read it but not reply. The majority (15/20, 75%) of 1000-day women also showed a high level of interest in interactive messaging that allowed them to reply to received text messages. They mentioned that they would respond to questions, reply with thanks, or provide feedback on the messages. Furthermore, nearly all (19/20, 95%) 1000-day participants reported that they would share information from the text messages with family members (eg, husband and mother-in-law), friends, and neighbors. Mothers-in-law from Kaligandaki, however, mentioned that when a daughter-in-law receives a text message, she would prefer not to share it with her mother-in-law because of differences in thoughts and attitudes. A health worker and a male decision-maker from Chapakot mentioned that they would like to share the text messages via Facebook to spread the word. Additionally, a health worker from Chapakot pointed out that sharing depends upon the nature and source of the message as follows:

If there is a message from Suaahara, you should also read. It was about what we should feed our children, and how should we take care of our children. We have to take care and feed the children.

Health facility staff, Chapakot, FGD

## Discussion

This study describes the interest, preferences, and skills related to a text message–based mHealth intervention among both 1000-day households and health and nutrition service providers. Our findings confirm high mobile phone ownership with sufficient skills among study participants to engage in a text message–based mHealth intervention. Participants preferred reminder messages that would provide information about the date and time of community-based services, such as HMGs. They also preferred informational text messages related to health and nutrition and interactive text messages from known phone numbers. The prioritized recipients were 1000-day women and FCHVs.

Prior research has shown promising use of text messages to improve uptake of health facility maternal and child health services in LMICs [5,6] and FCHV mobile phone use for data collection and disease surveillance following adequate training and support in Nepal [19,20]. Our study findings suggest that text messages could also be used to increase uptake of community health and nutrition services, such as HMGs meetings led by FCHVs, in Nepal and in similar low-resource settings.

Our study found that although 1000-day women have access to other phones in their families, they prefer to use their own phones and phone sharing is uncommon, which is important because previous studies have found reliance on shared phones a challenge for mHealth interventions. This is particularly true for retrieved voice messages [21] and for sensitive topics, such as family planning [22], because the information conveyed does not remain confidential [23]. Our findings suggest that information conveyed via an mHealth intervention in the hilly areas of Nepal will remain confidential and will reach the intended recipient. This highlights that phone sharing should be considered when designing mHealth interventions in LMICs [23].

Understanding target populations’ message delivery preferences is important [9] as mHealth interventions are more likely to be effective when message frequency, content, and style are tailored to these needs and preferences [15]. In our study, the majority of participants preferred receiving messages once a month and not early in the morning or at night. This is consistent with a qualitative study on text messaging preferences among adolescent females in the United States, where participants reported a desire for text messages in the afternoon or evening [24]. According to a meta-analysis, interventions using user-driven tailored message frequency were more beneficial compared with interventions that applied fixed or varying frequency [25].

In our study, we found a mixed preference for message delivery among health facility staff and FCHVs. Nearly half (5/10, 50%) of the FCHVs favored voice messages, whereas some FCHVs and health staff favored a combined approach of both text and voice messages. The preference of FCHVs for voice messages, given that they were slightly older and that some were illiterate, is consistent with findings from a cluster randomized controlled trial in the United States that found younger and higher-skilled users favored text messages [26]. Meanwhile, a study in rural Malawi determined that voice messages are more desirable by illiterate users [21]. These studies concluded that mHealth interventions need to include multiple modalities to ensure more participants benefit. This aligns with our findings and reflects that mHealth interventions in LMICs may be on the cusp of change owing to increased literacy levels.

We also found that although participants would read messages from unknown phone numbers, they would not value them. This suggests that before starting an mHealth intervention or sending text messages, programs should prepare the target audience and ensure the phone number to be used is communicated. Participants also expressed a strong interest in replying to text messages, suggesting that the mHealth intervention may be more acceptable if it requests a reply. This would also help to monitor whether messages have been received and read or to check whether messages were understood. Literature to date, however, points to contradictory findings on the benefits of this type of interaction. A meta-analysis on the efficacy of text messaging showed no difference between interventions that text participants and ask them to text back and those that text participants only [25]. Meanwhile, another systematic review of periodic messaging interventions showed that mHealth interventions using participants’ feedback were more effective compared with interventions not using feedback [27].

Owing to the purposive sampling of the data collection area, selection bias may have influenced the study results. Yet, when selecting study participants, we ensured diversity in age, education, caste, and ethnicity, so we believe our results reflect a broad range of people in the hilly areas of Nepal. The mountains and lowland plains, however, have not been reflected in this study. It is also important to note that the study was conducted in a *Suaahara II* implementation district, and thus, the study participants may have more exposure to study topics than populations residing in districts without at-scale health and nutrition interventions. We anticipate that if the intervention we designed can be successfully implemented and proven effective in improving health outcomes in the context of the *Suaahara II* program, it can be scaled up to additional geographies in Nepal. This would require additional validation of the findings across a broader range of settings beyond the one in this study. Furthermore, additional research is needed to deepen our understanding of the opportunities to use mHealth interventions to improve service reach and delivery in Nepal and other LMICs. For example, further implementation research on the effectiveness and costs associated with different implementation modalities (eg, text only, voice only, and combined messages as well as one-way versus interactive messaging) is needed.

Text messaging appears to be a promising cost-effective way to deliver health and nutrition information in remote and geographically dispersed populations like those in Nepal. Unlike other mHealth interventions, such as the use of applications, text messaging is easy to implement and does not require a special phone. This study provides important insights for the design of a text message–based mHealth intervention and suggests that mHealth has the potential to improve community health and nutrition service utilization in Nepal, particularly by sending meeting reminders and by providing information. Different groups had different preferences and requirements for potential text messages. Text message–based mHealth interventions in under-resourced settings could be encouraged and may result in higher service coverage and ultimately improved health and nutrition practices.

## Abbreviations

ANC: antenatal care
BCC: behavior change communication
FCHV: female community health volunteer
FGD: focus group discussion
FHI: Family Health International
HMG: health mothers’ group
IDI: in-depth interview
LMIC: low- and middle-income country
